# The triggering receptor expressed on myeloid cells (TREM) in inflammatory bowel disease pathogenesis

**DOI:** 10.1186/s12967-014-0293-z

**Published:** 2014-10-28

**Authors:** Marco Genua, Sergio Rutella, Carmen Correale, Silvio Danese

**Affiliations:** IBD Center, Humanitas Clinical and Research Hospital, Rozzano, Italy; Division of Translational Medicine, Research Branch, Sidra Medical & Research Center, Doha, Qatar

**Keywords:** Inflammatory bowel disease, TREM, Myeloid cells, Bacterial sensing, Inflammation, Innate immunity

## Abstract

The Triggering Receptors Expressed on Myeloid cells (TREM) are a family of cell-surface molecules that control inflammation, bone homeostasis, neurological development and blood coagulation. TREM-1 and TREM-2, the best-characterized receptors so far, play divergent roles in several infectious diseases. In the intestine, TREM-1 is highly expressed by macrophages, contributing to inflammatory bowel disease (IBD) pathogenesis. Contrary to current understanding, TREM-2 also promotes inflammation in IBD by fueling dendritic cell functions. This review will focus specifically on recent insights into the role of TREM proteins in IBD development, and discuss opportunities for novel treatment approaches.

## Introduction

Inflammatory bowel diseases (IBD) refer to chronic inflammatory disorders affecting the gastrointestinal tract, whose etiology is yet unknown. The main factors contributing to disease pathogenesis have recently been identified and among these, the gut microbiota, which is dominated by intestinal bacteria, is emerging as a key candidate for the design of innovative therapeutic strategies. Bacterial compounds are recognized by a variety of receptors, including the well-known Toll-Like Receptor (TLRs). The relationship between IBD pathogenesis and TLRs has been extensively studied leading to the discovery of several mechanisms and/or genetic alterations that drive intestinal inflammation. A new family of cell-surface receptors has recently emerged as a potential modulator of the inflammatory response. The Triggering Receptors Expressed on Myeloid cells (TREM) control several cell processes, including inflammation, bone homeostasis, neurological development and coagulation.

In this review, we will provide an overview of TREM gene clusters focusing on the expression of TREM family members and their role in inflammation. We will then analyze in detail both TREM-1 and TREM-2 signaling and regulation, with emphasis on the cell-type specificity of their action. Recent insights on their activity in IBD pathogenesis reinforce the opportunity to control TREM functions as a new therapeutic approach.

### TREM: a novel class of immune receptors

Innate inflammatory responses are essential for the body to defend itself against pathogens. A fine-tuning of immune reactivity is, however, crucial to prevent excessive inflammation and ensuing tissue damage. Pathogen sensing is guaranteed by pattern recognition receptors (PRRs), which identify specific pathogens, activate innate immune responses and shape adaptive immunity. Recently, another class of cell-surface receptors has been identified: Triggering Receptor Expressed on Myeloid cells (TREM). These receptors are important regulators of the immune response, due to their ability to either amplify or decrease PRR-induced signals.

#### Human and mouse gene clusters

The identification of the first member of the TREM family, TREM-1, was achieved through research on the overlap between cDNA and the nucleotide sequence encoding for the NKp44 polypeptide on the GeneBank EST database [1]. This research led to the recognition of a novel gene cluster comprising several activating receptors within the IgG superfamily. In addition, TREM-1’s open reading frame (ORF) matched to one related sequence, referred to as TREM-2 [1]. Subsequently, TREM-2 was also identified and characterized in a murine macrophage cell line, and compared to the human gene [2].

**Figure 1:**
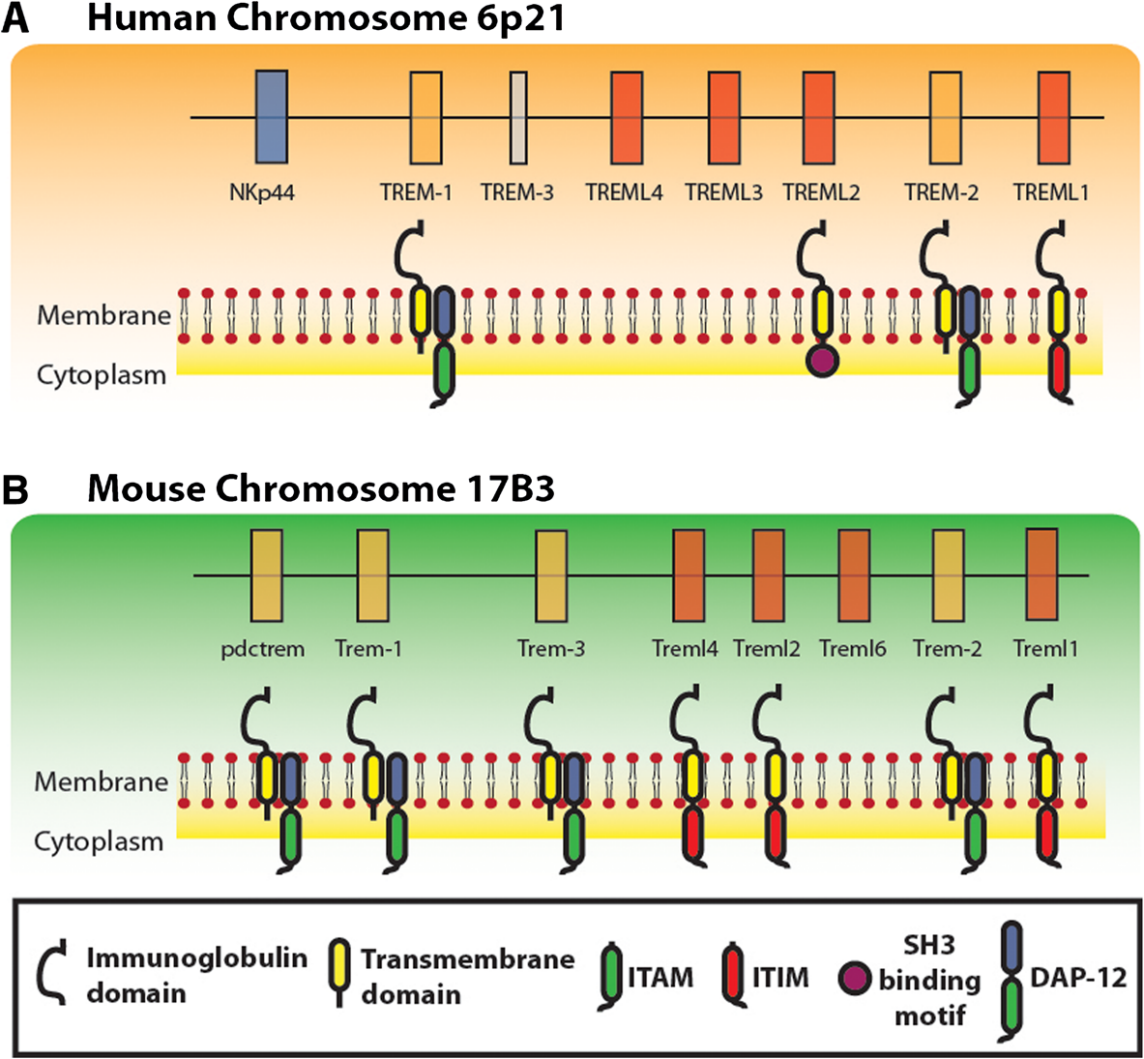
**The TREM gene cluster in mice and humans.** Schematic diagram that compares the order of genes in the TREM clusters in humans **(A)** and mice **(B)**. Genes encoding TREM protein were drawn in orange, whereas TREM-like genes are depicted in red. Models of the encoded proteins are shown below the corresponding genes. The DAP12 adaptor is also shown with its ITAM. TLT proteins are shown with the corresponding ITIM.

Currently, the human TREM gene cluster (Figure 1A) includes several other members, namely the TREM-like transcripts −1, −2, -3, and −4 (TREML1, TREML2, TREML3 and TREML4). The murine gene cluster (Figure 1B) however did not include NKp44, but did contain genes encoding for the plasmacytoid-dendritic cell Trem (pDC-Trem), Treml −1, −2, −4 and −6, and Trem3. This latter gene was found also in the human cluster as a non-transcriptionally active pseudo-gene [3]. It is important to note that while both the murine and human gene clusters cover many putative coding sequences (reviewed in [4], Figure 1), protein products have been discovered and characterized only for few genes, as discussed below.

#### TREM structural and functional properties

TREMs share common structural properties, including the presence of a single extracellular immunoglobulin-like domain of the V-type, a trans-membrane domain and a short cytoplasmic tail (Figure 1). In particular, the trans-membrane domain possesses negatively charged residues that interact with the positively charged residues of the DNAX Activating Protein of 12 kDa (DAP12), a trans-membrane adaptor containing an immunoreceptor tyrosine-based activation motif (ITAM). The TREM like transcript −1 (TLT-1), whether of human or murine origin, instead contains an intracellular domain with an immunoreceptor tyrosine-based inhibitory motif (ITIM) in the cytoplasmic tail, directly controlling downstream signaling [5]. Similarly, TLT-2 of murine origin contains an ITIM, whereas human TLT-2 possesses a putative SH3 binding motif [4,6] (Figure 1).

The extracellular Ig-like domain is responsible for the recognition and binding of molecules. Unfortunately, data available in current literature does not address the identification of neither TREM-1 nor TREM-2 endogenous ligands. The first evidence of these ligands stems from Gibot and Colleagues, who suggested the presence of a TREM-1 ligand in the surface of neutrophils during endotoxemia [7]. Subsequently, a TREM-1 ligand was recognized in the surface of platelets, both in resting condition and after thrombin activation [8]. Furthermore, recent studies by Derive and Colleagues suggested that the endogenous ligand for TREM-1 competes with a 17 aminoacid sequence obtained from the cleavage of the extracellular domain of TLT-1 [9]. As for TREM-1, TREM-2Fc/chimera stitched on the surface of bone marrow-derived cells, suggesting the presence of a putative ligand on their surface [10]. Similarly, ligands have been identified in the anionic residues present on the surface of GRAM positive and negative bacteria [11]. Finally, Piccio and Colleagues found that the astrocytoma cell line HTB12 expresses a ligand for TREM-2 [12].

At present, the only fully characterized ligand is the membrane protein B7-H3, a member of the B7 family, which binds TLT-2. Interestingly, a different strategy was used to identify TLT-2 ligands, by searching for homologues of CD28 family members, known receptors of B7 proteins [13]. It will be useful to employ similar novel bio-informatic strategies in future to pursue the identification of TREM ligands, especially given their peculiar ability to interact with a broad spectrum of stimuli, instead of recognizing a specific pathogen.

An additional important feature of TREM proteins is their release as soluble forms (sTREM). Soluble TREM may in fact be generated by the alternative splicing of mRNA, producing secreted receptor isoforms [14]. Otherwise, sTREM may be produced by the cleavage of the extracellular domain off the cell surface [15]. To date, the predominant source of sTREM remains unknown; however, DAP12-coupled (TREM-1 and TREM-2) and non-DAP12-coupled (TLT-1) soluble receptors have been identified in biological fluids of both animals and patients with inflammatory and infectious disorders [16-19], suggesting their importance as new biological markers. In general, soluble forms of receptors, such as sTREM-1 and sTREM-2, seem to negatively regulate TREM signaling through neutralization of the respective ligands, acting as decoy receptors [20,21].

In summary, the structural and functional features of TREM proteins support their involvement in controlling immune response.

### Expression pattern and functions of TREM family members

Cells of the myeloid lineage primarily express TREM proteins [4,22,23], but TREM have further been detected in other cell types, such as platelets [24], bronchial epithelial cells [25], gastric epithelial cells [26], and hepatic endothelial cells [27].

Among the putative genes, human TREM1, TREM2, TREML1 and TREML2, together with their correspondent murine versions, have all been shown to translate into protein products [22]. Moreover, proteins encoded by the murine genes Trem3 [3], pDC-Trem [28], and Treml4 [29] have also been detected.

TREM-1, which remains the most studied receptor within its family, was initially identified in differentiated monocytes and in circulating blood neutrophils [1]. Subsequently, TREM-1 was detected in macrophages of both alveolar [30], hepatic [27], and intestinal origin [31,32]. Recently, TREM-1 expression has also been reported in endothelial cells [27] and gastric epithelial cells [26]. While TREM-1 is recognized as an amplifier of inflammation in cells of myeloid origin, its expression and activities in endothelial and epithelial cells require further study.

TREM-2 was instead first detected on human monocyte-derived dendritic cells (DC) [2,33], following which its expression was revealed in osteoclast and microglia cells [34], as well as in alveolar [35], hepatic [27] and intestinal macrophages [36]. Similarly to TREM-1, TREM-2’s expression was also detected in endothelial cells [27] although its function in this cell type is not yet fully characterized. Conversely to TREM-1, TREM-2 expression and function have been linked to anti-inflammatory activities, especially in the central nervous system (CNS), where TREM-2 expression is required for the differentiation of myelinating oligodendrocytes [37].

Among the other TREM family members, Trem-3 has been detected in mouse macrophages, where it is up-regulated after lipopolysaccharide (LPS) exposure [3]. Similarly, pDC-Trem has been detected in plasmacytoid DCs of murine origin, after CpG stimulation [28]. Interestingly, Treml4 has been recently studied on mouse splenic DCs, revealing its importance in recognizing apoptotic and necrotic cells [29]. Finally, TLT-1 was initially identified in human platelets and megakaryocytes [38], where it counteracted TREM-1 function, whereas TLT-2 has been located in B and T-cell lines of human origin, as well as in murine neutrophils and macrophages [6].

While TREM-1 and TREM-2 have been extensively studied and characterized in several pathological conditions, reports regarding the expression and function of the other TREM are just at the beginning. Therefore, additional information is required to better understand the relevance and the significance of these receptors both under physiological conditions and in disease states.

### The dual-face of TREM-1 and TREM-2 in inflammation

Despite the conventional view, which dictates that TREM-1 is a pro-inflammatory mediator whereas TREM-2 mainly dampens inflammatory responses, recent evidence has shed light into the complex and multi-faceted role of these receptors.

Initial findings established TREM-1 as an amplifier of inflammatory responses in experimental sepsis [39]. During infection, receptor expression is modulated and soluble TREM-1 is released [40,41]. In addition, recombinant TREM-1/Fc fusion proteins or antagonistic peptides rescue mice from endotoxemia or polymicrobial sepsis [42].

Despite the apparent detrimental effects of overstimulation through TREM-1, its signaling may also be required for protective anti-inflammatory responses. It has been shown that a moderate dose of TREM-1 siRNA improves mice survival during polymicrobial sepsis, whereas high-dose siRNA leads to full silencing of TREM-1, blunting neutrophil respiratory bursts and increasing mortality in mice [43]. In contrast to polymicrobial sepsis, full TREM-1 silencing has been shown to play a protective role in endotoxemia [43]. Mice treated with an agonistic TREM-1 antibody and infected with S. *pneumoniae* exhibited an enhanced induction of the early inflammatory response. However, prolonged activation of TREM-1 did promote the resolution of pneumonia, leading to an accelerated elimination of bacteria, and consequently improved survival [44].

In agreement with these findings, TREM-1 has been recently linked to trans-epithelial migration of neutrophils after infection with P. *aeruginosa* [45]. TREM-1/3 deficiency resulted in an unexpected increase of local and systemic cytokine production. In fact, neutrophils lacking TREM-1/3 retained unaltered bacterial killing, via phagocytosis and chemotaxis; however, histological examination of TREM-1/3-deficient lungs revealed decreased neutrophil infiltration of the airways [45].

Since results obtained to date on TREM-1’s function in controlling microbial infection have proven controversial, novel tools are required to unambiguously investigate its function. To this extent, two different groups have recently generated TREM-1 deficient mice [46,47], with Weber and Colleagues demonstrating that TREM-1 genetic deletion ameliorates dextran sulfate sodium (DSS)-induced colitis while it does not appear to affect control of the infection by specific pathogens, such as *Leishmania Major*, influenza virus and *Legionella pneumoniae*, displaying however a reduced neutrophil infiltration [46]. TREM-1 KO mice have likewise been studied in a model of *Klebsiella pneumoniae* liver abscess (KPLA) [48]. Interesting, the absence of TREM-1 increased *K. pneumoniae* dissemination, enhanced liver and systemic inflammation, and reduced survival, as a result of impaired bacterial clearance in the small intestine [48]. We may thus conclude that TREM-1’s function and activities may be dependent on the type of infection.

TREM-2 regulates the differentiation of a broad range of myeloid cells, including osteoclasts and oligodendrocytes. In fact, patients with Nasu-Hakola disease (NHD) characterized by pre-senile dementia and bone cyst formation, harbor loss-of-function mutations in the genes encoding DAP12 and TREM-2 [49]. Both genetic lesions lead to the development of an identical disease, resulting from the failure of TREM-2 signaling due to a defect in either TREM-2 itself or the associated DAP12 signaling cascade.

The detrimental effect of TREM-2 abrogation in the CNS is also supported by the study of the experimental autoimmune encephalomyelitis (EAE), an animal model for multiple sclerosis. TREM-2 expression is up-regulated on microglia cells and infiltrating macrophages in both the spinal cord and brain during inflammatory burst [12], and myeloid cells transduced with TREM-2 dampen EAE in mice, by migrating to the CNS and releasing anti-inflammatory cytokines [50].

Contrary to current understanding, recent work by Correale et al. [51] and Sieber et al. [52] provided evidence that, in experimental models of gut inflammation and stroke, loss of TREM-2 function leads to an attenuated inflammatory reaction. The absence of TREM-2 in intestinal lamina-propria DCs was likewise shown to lead to a reduced production of pro-inflammatory cytokines and mitigation of T-cell activation during experimental colitis [51]. Similarly, genetic deletion of TREM-2 leads to a decreased production of TNF-α, IL-1β and IL-6 after stroke, associated with reduced microglia activation [52].

Further studies are needed to better elucidate the functions and activities of TREM-1 and TREM-2, especially in regards to single-pathogen infection as compared to a polymicrobial-driven disease. Evaluating the signaling elicited by TREM-1 or by TREM-2 may represent a winning strategy to clarify their function.

### The cell-type specificity of TREM-1 and TREM-2 elicited signaling

Literature on TREM-1 signaling has been recently reviewed [23]. Signaling cascades activated by the interaction between TREM-2 and DAP12 have been addressed by Paradowska-Gorycka and Jurkowska [53], and readers are referred to those papers for a thorough review of these issues.

**Figure 2:**
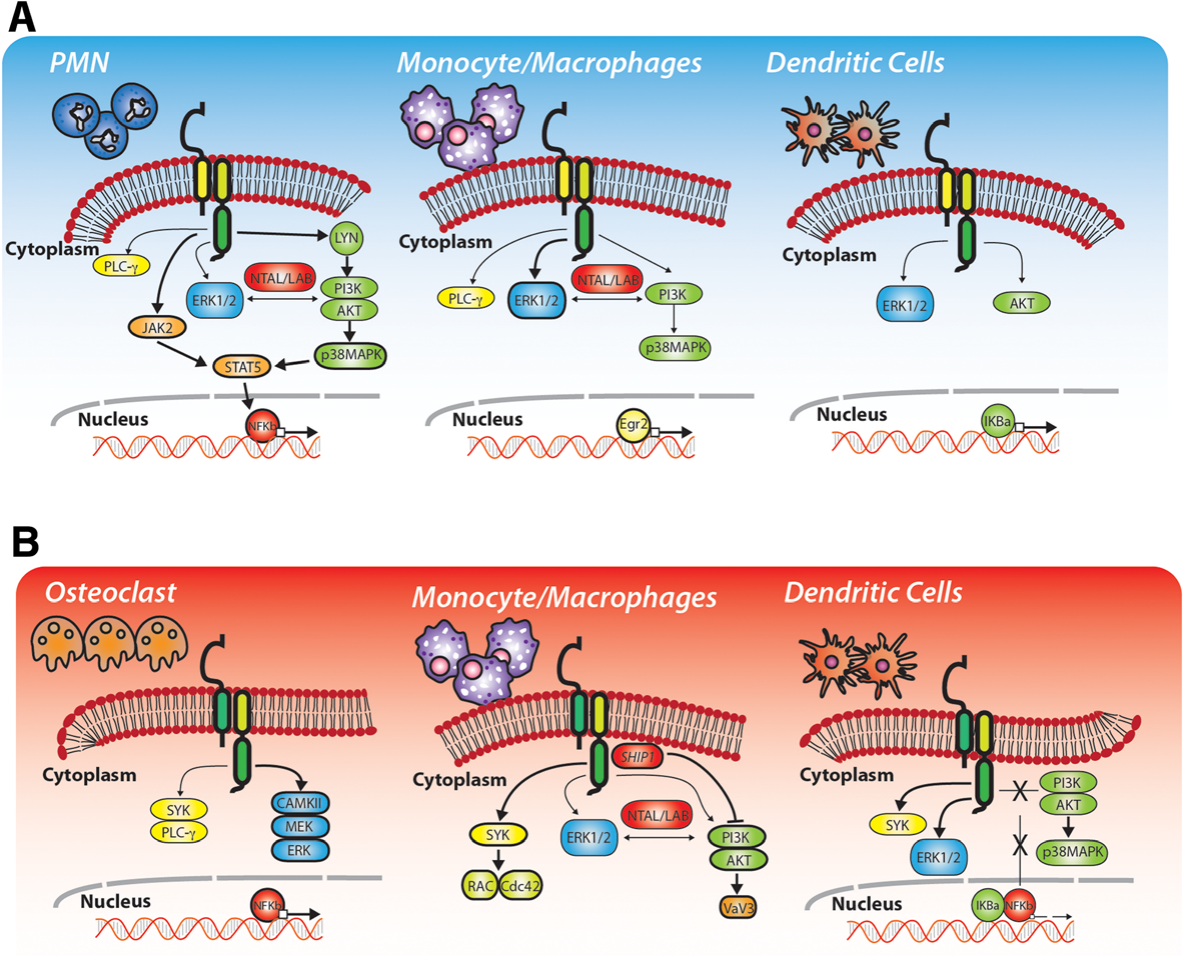
**TREM-1 and TREM-2 signaling.** Overview of the main intracellular mediators activated by TREM-1 **(A)** and TREM-2 **(B)**, in order to elicit downstream signaling. Depending on the cell-type involved, TREM-1 or TREM-2 ligation might activate different cytoplasmic adaptors, producing different outcomes.

The use of cross-linked antibodies or fusion proteins has been the main strategy pursued to date to identify intracellular mediators and signaling cascades, activated either by TREM-1 or by TREM-2. In this work we will systematically review the main intracellular mediators and the principal outcomes directly linked to TREM-1 or TREM-2 activation, specifically focusing on the cell types involved in these responses (Figure 2).

#### Neutrophils

Neutrophils are essential for phagocytosis and proteolysis through which bacteria, cellular debris and particles are removed and destroyed. Therefore, neutrophil function and activities are crucial during a poly-microbial systemic infection, such as sepsis. Interestingly, TREM-1 is specifically enhanced in neutrophils during sepsis [1], while TREM-2 expression was not detected in this specific cell type.

- Intracellular mediators
  TREM-1 engagement was initially related to the activation of the extracellular signal-related kinase 1/2 (ERK1/2), and phospholipase C-γ (PLC-γ) [1]. Subsequently, studies from Tessarz and Colleagues [54] clarified that TREM-1 is also able to couple with the non-T cell activation linker (NTAL, also called linker of activation in B cell, LAB). TREM-1/NTAL interaction reduces ERK1/2 phosphorylation and Ca^2+^ mobilization. The complex picture of TREM-1’s signaling cascade includes the tyrosine-protein kinase Lyn, the Proteinase Kinase B (AKT), and the Janus Kinase-2 (JAK2). JAK2 activation ultimately leads to the translocation of STAT5 and RelA, a subunit of the nuclear factor-κB [55]. Finally, Haselmayer and Colleagues showed that TREM-1 ligation activates the phosphatidylinositol-4,5-bisphosphate 3-kinase (PI3K), and the mitogen-activated kinase p38MAPK [56].
- Outcomes
  The first effect elicited by TREM-1 ligation is a DAP-12 dependent Ca^2+^ influx [1]. This event is followed by the activation of signaling cascades with Jak/STAT and NF-κΒ as main elements [55]; TREM-1 consecutively promotes the release of several pro-inflammatory cytokines, such as IL-1β, IL-2, IL-6, IL-8, IL-12p40, and TNF, as well as chemokines, such as macrophage inflammatory protein (MIP)-1α [54,57-59]. As a final outcome, TREM-1 activation increases neutrophil degranulation and phagocytic activity.

#### Monocyte/Macrophages

Macrophages are specialized immune cells, strategically stationed as “sentinels” where microbial invasion or accumulation of exogenous particles occurs. Under pathological conditions, circulating monocytes are recruited to the injured or inflamed tissues, where they differentiate into specialized macrophages, such as Kupffer cells in the liver, osteoclasts in the bone, and microglia cells in the CNS. While we observed a specific expression of TREM-1 in neutrophils, monocytes and macrophages co-express TREM-1 and TREM-2. However, current literature has not explored the possible cross-talk between the two receptors.

- Intracellular mediators
  As for neutrophils, TREM-1 activation in monocytes/macrophages leads to the activation of ERK1/2 and PI3K [1]. Liver macrophages have been also studied after LPS stimulation, in the presence or absence of the TREM-1/Fc fusion protein, confirming that the downstream activation of PI3K and p38MAPK is dependent on TREM-1 [27]. Interestingly, a recent work from Yuan and Colleagues took advantage of the availability of TREM-1 deficient macrophages and of TREM-1 over-expressing adenovirus, proving that TREM-1 signaling is involved in the activation of the transcription factor early growth response protein 2 (Egr2) [60].
  In parallel, TREM-2 signaling has been also evaluated in macrophages, where it was found to be essential for the activation of SYK, and involved downstream both the small guanosine triphosphatases RAC1 and Cdc42, leading ultimately to actin-dependent cytoskeleton rearrangement [61]. Moreover, a recent study from Peng and Colleagues further explored TREM-2 induced signaling in macrophages and osteoclasts, clarifying that Ca^2+^ influx is also mediated by the activation of PI3K, ERK1/2 and the guanine nucleotide exchange factor Vav3; these activities required the adaptor molecule DAP10 and were inhibited by the Src homology 2 (SH2) domain-containing inositol phosphatase-1 (SHIP1) [62]. Studies on osteoclastogenesis have proven that TREM-2 is implicated in Ca^2+^-mediated activation of the receptor activator of NF-κB (RANK), which is required to promote downstream activation of calcium/calmodulin-dependent protein kinase (CaMK)II, MEK and ERK [63]. Finally, NTAL engagement is also crucial in TREM-2 induced signaling in macrophages, since it has been shown to regulate monocyte-to-macrophages differentiation, controlling subsequent anti-inflammatory responses [64].
- Outcomes
  A complete picture of the outcomes elicited by TREM-1 or TREM-2 activation in monocyte/macrophages is the subject of intense investigation. The currently held view recognizes TREM-1 as a skewed pro-inflammatory receptor, which augments the release of cytokines, such as TNF, IL-6, IL-8 and IL-1β [32]. Moreover, TREM-1 up-regulation has been found in Tumor-Associated Macrophages (TAM) isolated from patients with lung cancer [65]. Conversely, TREM-2 expression is necessary to promote the differentiation of osteoclasts [34], as to accelerate intestinal wound healing mediated by macrophage polarization [36].
  At present it remains unclear whether the differences observed are due to a TREM-specific recruitment of intracellular mediators, or to a different synergism with other immune receptors, such as TLRs. Moreover, recent insights have underlined the importance of taking into account the expression levels of other innate immune receptors, and the complexity of the interaction between the environment and those receptors, especially in macrophages from different tissues [66].

#### DCs

DCs are professional antigen-presenting cells interspersed within tissues, and are also in close contact with the external environment, as such representing the link between the innate and adaptive immune system. Interestingly, TREM-1 and TREM-2 are both expressed by DCs, although TREM-2 was the first to be recognized as a central player in DC maturation and activation [33].

- Intracellular mediators
  While TREM-1 signaling has been widely studied in neutrophils and macrophages, only one study attempted to identify the effect of TREM-1 activation in mature DCs, clarifying that receptor engagement led to the activation of ERK-1, Protein Kinase B (AKT), and the transcription factor IκBα [67].
  Conversely, TREM-2 was initially investigated during DC maturation, and therefore analyses of the intracellular mediators focused on these specific cell-types. In particular, in monocyte-derived DCs, TREM-2 ligation led to a rapid rise in the intracellular level of Ca^2+^, followed by the activation of Protein Tyrosin Kinases (PTKs), such as SYK, and ERK1/2, without inducing IκBα/NF-κB or p38/MAPK signaling pathways [33].
- Outcomes
  The discovery that TREM-1 is also implicated in the control of DC function is very recent, with Bosco and Colleagues having identified TREM-1 as a hypoxia-inducible gene [67]. Their work provided evidence that TREM-1 engagement promotes the up-regulation of T-cell co-stimulatory molecules and homing chemokine receptors, typical of mature DCs, as well as increasing the production of pro-inflammatory stimuli, in turn provoking intensified Th1/Th17-cell responses [68].
  Similarly, TREM-2 activation in DCs leads to enhanced expression of differentiation markers, such as CCR7 [33]. However, controversial results have been reported regarding the outcomes elicited by TREM-2 activation. In fact, Ito and Colleagues analyzed the effects of TREM-2 genetic deletion on bone marrow-derived DCs, showing increased release of TLR-induced pro-inflammatory cytokines [69], in sharp contrast with TREM-2^−/−^ intestinal DCs which display lower production of pro-inflammatory cytokines in response to TLR ligands [51].

#### TREM and PRRs liaison: effects on the elicited signaling

While studies conducted by cross-linking antibodies have been instrumental in the identification of several intracellular mediators, further research is necessary to clarify the function of TREM, taking into account the cell types involved and the interacting environment.

Among the factors that may contribute to TREM differential responses, the expression pattern of PRRs may be crucially involved in the cell type specificity of the inflammatory response. Remarkably, a comment from Julia Klesney-Tait and Marco Colonna briefly revised the interconnection between TREM-1 and TLR [70], analyzing in detail the report from Ornatowska and Colleagues [71]. Using pathway-specific microarray analyses, the authors clarified that TREM-1’s synergism with TLR4 is dependent on the intracellular molecular adaptor engaged [71]. In fact, in macrophages silenced for TREM-1 by RNA interfering, the signaling triggered by TLR4 through the engagement of TIR-domain-containing adapter-inducing interferon-β (TRIF) is not affected. In contrast, MyD88 and CD14 transcripts were decreased following LPS stimulation in the setting of TREM-1 silencing [71].

Similarly, TLR engagement has also been evaluated in the context of DCs, providing evidence that TREM-2-deficient BMDCs had increased TLR-induced maturation and showed increased production of inflammatory cytokines [69]. However, opposing results were obtained when TREM-2’s deficiency effect was evaluated in intestinal DCs [51].

In line with this data, the expression profile of selected innate immune receptors has been outlined among the various myeloid cell types [66], clarifying that a fine-tuned crosstalk between such receptors is necessary to promote anti-microbial defenses and avoid immune-related pathologies [66]. A far greater effort is therefore necessary to unveil the expression and functional profile of TREM proteins in the different subset of myeloid cells.

### TREM-1 and TREM-2: friend or foe in IBD?

IBD refers to chronic inflammatory disorders that affect the gastrointestinal tract [72]. There are two main clinical forms of IBD, namely Crohn’s disease (CD) which can affect any part of the intestine, and Ulcerative Colitis (UC) which is restricted to the colonic mucosa [73]. Although the pathogenesis of IBD is not yet fully understood, it is becoming evident that these chronic disorders affect genetically susceptible patients, leading to an increased exposure of immune cells within the intestinal lamina-propria to luminal bacteria [74].

**Figure 3:**
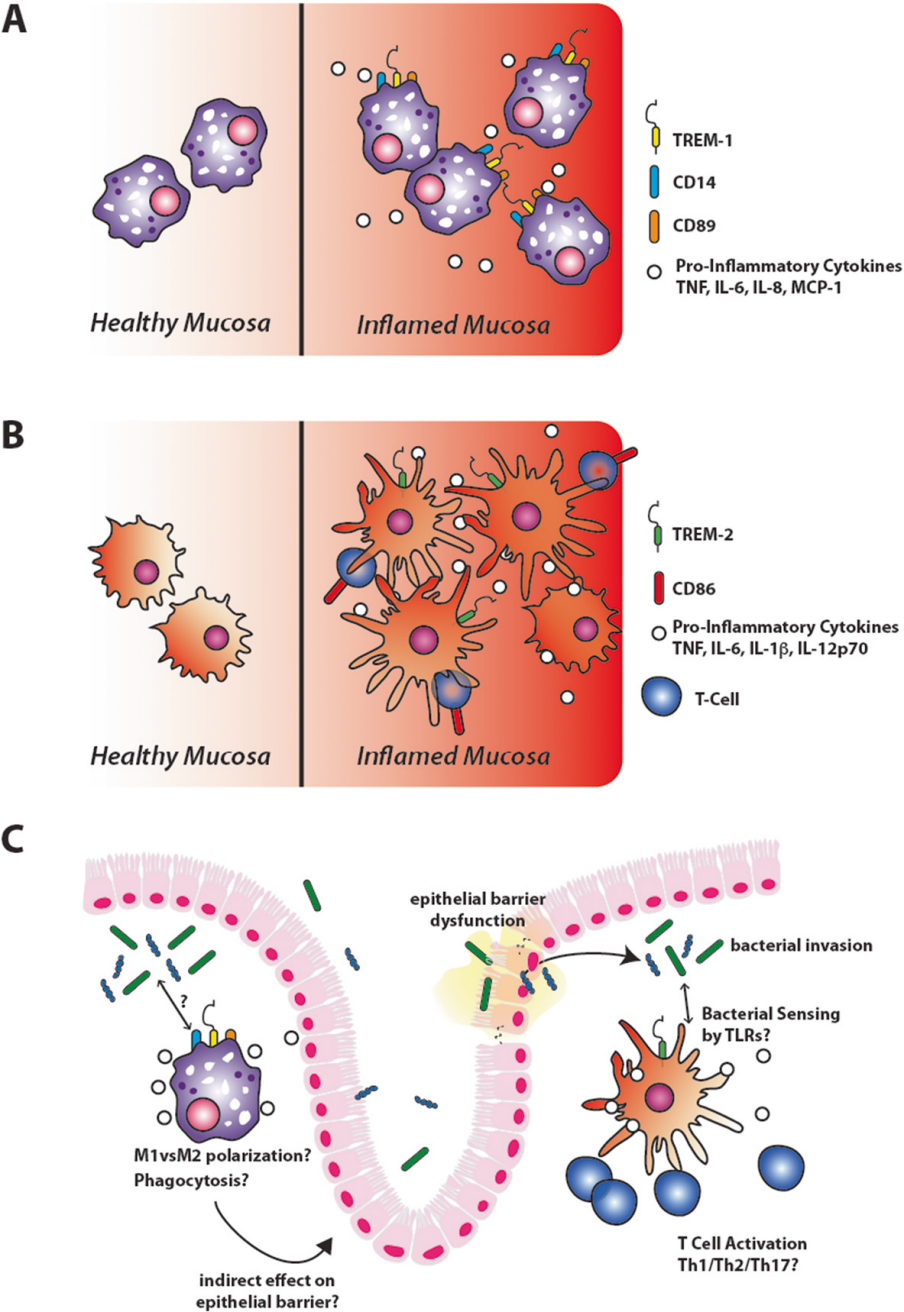
**TREM-1 and TREM-2 role in IBD.** TREM-1 and TREM-2 exert similar activities in the gut microenvironment leading to the amplification of intestinal inflammation. **(A)**, TREM-1 is expressed on the macrophage surface of IBD patients leading to the increased production of TNF-α, IL-6, IL-8 and MCP-1. **(B)**, TREM-2 is expressed on the surface of mature DC exclusively in the inflamed mucosa. Increased expression of TREM-2 drives the production of pro-inflammatory cytokines, such as TNF-α, IL-6, IL-1β and IL-12p70, and induces T-cell activation. **(C)**, It’s still unknown if in the inflamed mucosa of IBD patients, TREM-1 positive macrophages possess different phagocytic or killing activity, or if they displayed a peculiar polarization. Similarly, it's not known if the pathogenic role of TREM-2 positive DCs could be mediated by selective interaction with specific TLR.

Due to the peculiar activities of TREM-1 and TREM-2 in controlling bacterial infection, the functional role of these two receptors has also been studied in IBD patients and in experimental models of colitis (Figure 3).

#### TREM-1 in IBD: could it be a therapeutic target in the near future?

In both a T-cell transfer model of colitis in RAG2^−/−^ mice and in C57BL/6 mice with DSS-induced colitis, TREM-1 mRNA and protein have been shown to be significantly up-regulated, a finding that preceded the appearance of histological signs of the disease. Furthermore, administration of an antagonist TREM-1 peptide (LP17), even after the establishment of colitis, decreased disease severity as evaluated by the extent of colon shortening and by milder alterations in colon histopathology [32]. LP17 treatment also reduced TNF-α tissue levels. These findings uncovered an important role for TREM-1 in the establishment of IBD and suggest that targeting TREM-1 in the intestine could be beneficial for patients suffering from IBD. While only few TREM-1-expressing recently recruited blood monocytes can be found in the normal colon, TREM-1 positive macrophages can be readily detected in the lamina propria of patients with IBD, with no apparent differences between active CD and UC [32]. TREM-1-expressing macrophages in gut biopsies of patients with IBD display a pro-inflammatory phenotype, consisting of CD14 and CD89 co-expression. Therefore, TREM-1-expressing macrophages acquire an aberrant phenotype in IBD, and in vitro engagement of TREM-1 with a cross-linking antibody resulted in enhanced production of TNF-α, IL-6, IL-8, and monocyte chemotactic protein (MCP)-1 (Figure 3A) [32]. The origin of TREM-1-expressing macrophages in IBD patients however remains a matter of debate, since the concomitant expression of CD14 and CD89 may suggest that the macrophages are exudative cells recruited to the inflamed gut. In agreement with these results, a recent work by Weber and Colleagues indicated that, in the murine intestine, TREM-1 is prevalently expressed by a pro-inflammatory sub-population of macrophages [75]. TREM-1 inhibition through the administration of LP-17 has also been applied at the beginning of following the induction of experimental colitis and colitis associated carcinogenesis, revealing that the blockage of TREM-1 activity exerts anti-inflammatory properties while also diminishing epithelial proliferation [76]. Moreover, in the T cell-transfer and DSS-induced models of colitis, TREM-1 KO mice displayed a significantly attenuated disease, associated with reduced inflammatory infiltrates and diminished expression of pro-inflammatory cytokines [46].

In addition to these findings, soluble TREM-1 (sTREM-1) have been quantified in patients with IBD with the aim at establishing whether its serum levels could serve as surrogate marker of disease activity [77]. Both sTREM-1 and tissue TREM-1 mRNA were evaluated in patients with CD and UC enrolled in the Swiss IBD cohort study [77]. TREM-1 was significantly more expressed in gut tissues from patients with active disease whereas sTREM-1 was similarly elevated in patients with either active or inactive disease, although its levels were globally increased in patients compared with healthy controls. Studies in mice with colitis complemented these findings by showing that gut levels of TREM-1 mRNA directly correlated with disease activity, with increased sTREM-1 levels further measured in mice with more controllable disease. Other reports also documented an increase of sTREM-1 in patients with IBD, in close correlation with TNF-α and with disease activity, especially in patients with UC [78,79]. In particular, a total of 31 patients with UC and 22 patients with CD were evaluated for sTREM-1 levels. Mean sTREM-1 levels were significantly higher in IBD patients than in healthy controls [78]. Moreover, in UC patients the correlation between sTREM-1 levels and disease activity was much more robust than other parameters, such as erythrocyte sedimentation and or C-reactive protein [78]. Soluble TREM-1 has also been correlated with the occurrence of endoscopic markers of disease activity in a cohort of 58 patients comprising 19 healthy volunteers, 8 patients with CD and 31 with UC [80]. In this study sTREM-1 levels correlated with disease activity in CD patients, and with clinical and endoscopic disease activity index of ulcerative colitis patients [78,79].

Finally, it has been proposed that TREM-1 genetic polymorphisms may correlate with IBD development. Three TREM-1 single nucleotide polymorphisms (SNPs, rs2234237, rs3789205, and rs9471535) were recently evaluated in 202 Korean patients with CD, 265 with UC, 138 with intestinal Behcet’s disease (BD), and 234 healthy controls [80]. While TREM-1 SNPs were associated with the development of BD, no significant correlation was found within IBD patients [80].

#### The controversial role of TREM-2 in IBD pathogenesis

TREM-2 genetic variation has been recently correlated with Alzheimer’ disease (AD) [81]. However, it is still unclear how TREM-2 participates in AD pathogenesis, and whether TREM-2 blockade could be a therapeutic avenue [82]. Despite the evidence pointing to TREM-2 as an anti-inflammatory receptor, especially in the CNS, studies regarding its role in the intestine have yielded diverging results. In a model of mucosal wound healing, TREM-2 was required for efficient mucosal repair [36]. In fact, TREM-2^+^ infiltrating macrophages showed an increased expression of IL-4 and IL-13, required to properly restore epithelial wounds. Interestingly, TREM-2 KO mice displayed slow and incomplete wound healing accompanied with reduced epithelial proliferation and increased infiltrate of M1 macrophages [36]. Different results were obtained when the role of TREM-2 was addressed during IBD pathogenesis. In our study, TREM-2 was virtually absent from colon samples of control patients, but its levels were significantly higher in the inflamed mucosa of patients with IBD, where TREM-2 is mainly expressed by CD11c^+^ DCs [51] (Figure 3B). In line with this data, we found that TREM-2 genetic deletion led to a reduced susceptibility to experimental colitis. Similarly, mucosal levels of TREM-2 increased as acute or chronic colitis was induced in mice and furthermore, TREM-2 KO mice developed less severe colitis than wild-type mice, with a significantly lower decrease in body weight, lower disease activity index, and smaller mucosal lesions at endoscopic analysis. We also investigated colon DCs phenotypically and functionally, providing evidence that, in the absence of TREM-2, DCs produced lower levels of inflammatory cytokines, had reduced levels of bacterial killing and were defective in inducing T-cell activation in comparison with DCs from wild-type mice [51].

These findings highlight that even if TREM-2 possess an important role in microglia activation in the CNS, its role in the establishment of IBD remains controversial and, similarly to TREM-1, suggest that targeting TREM-2 locally in the intestine could be beneficial to treat IBD. As for TREM-1, further studies are needed in order to evaluate the mechanisms of regulation of TREM-2 in intestinal DCs as well as to identify a possible cross-talk with TLRs.

#### TREM-1 and TREM-2 in IBD: what we do not know

Despite emerging evidence and preclinical studies to date clearly supporting TREM-1 and TREM-2 as good candidates for therapeutic intervention, no clinical studies have yet been run with IBD patients.

As discussed above, observational studies correlated sTREM-1 levels with TNF levels in UC patients [78,79], but sTREM-1 may have limited use as a disease biomarker, since there appears to be no difference in sTREM-1 levels when comparing active and inactive disease [77]. It is therefore necessary to better define the molecular mechanisms of TREM-1 activities during IBD pathogenesis. A functional characterization of TREM-1 expressing macrophages is therefore needed in order to clarify whether they posses a peculiar polarization or whether they have an impaired capacity to control bacterial invasion (Figure 3C). Moreover, the relevance of other bacterial sensors, such as PRRs and, in particular, TLRs is still unknown.

Similarly, TREM-2 activity during IBD pathogenesis requires more extensive investigation. The finding that TREM-2^+^ DCs possess a pro-inflammatory phenotype does not apparently explain the increased percentage of this specific cell type in IBD patients. Similarly to TREM-1, the relevance of TLR activities is still unknown, even if it has been clarified that TREM-2 augments the production of pro-inflammatory cytokines after TLR engagement in the intestinal microenvironment [51]. Moreover, it will be essential to understand how TREM-2 DCs influence T- cell activation and proliferation (Figure 3C). Interestingly, while genetic models have been employed to analyze the role of both TREM-1 and TREM-2 in experimental IBD pathogenesis, the question whether genetic deletion of TREM-1 or TREM-2 alters the expression of the cognate receptor in the intestinal microenvironment had not yet been addressed.

## Conclusion

Myeloid cell function is closely controlled by members of the TREM family. While TREM-1 plays a role in the amplification of host inflammatory responses, TREM-2 may counterbalance the effects of TREM-1, being more broadly implicated in the regulation of bone physiology and microglia cell functions. The interactions between TREM receptors and the local microenvironment are therefore crucial to determine the outcome of an inflammatory response. A thorough characterization of the intracellular signaling pathways activated by TREM-1 and TREM-2 and the identification of cooperative interactions between TREM-1 and TREM-2 will allow for a better understanding of the role of these receptors in inflammatory disorders.

Recent advances regarding the peculiar role of TREM-1 and TREM-2 in modulating intestinal inflammation reinforce the opportunity to control their functions as a novel therapeutic approach. In particular, the administration of LP17 peptide showed promising results, especially related to the local inhibition of TREM-1 function in the intestine. Similarly, the recent association between increased mucosal levels of TREM-2 and IBD opens new avenues for clinical research. TREM-2 may also be implicated in the modulation of bacterial killing by DC, further defining TREM proteins as versatile modifiers of disease.
